# Epidemiological and microbiome associations of *Clostridioides difficile* carriage in infancy and early childhood

**DOI:** 10.1080/19490976.2023.2203969

**Published:** 2023-04-25

**Authors:** Jyoti Mani, Shira Levy, Angelina Angelova, Sahel Hazrati, Ryan Fassnacht, Poorani Subramanian, Tiana Richards, John E. Niederhuber, George L. Maxwell, Suchitra K. Hourigan

**Affiliations:** a Department of Pediatrics, Children’s National Medical Center, Washington, DC, USA; b National Institute of Allergy and Infectious Diseases, National Institute of Health, Bethesda, MD, USA; c Women’s Service Line, Inova Health System, Falls Church, VA, USA; d Inova Children’s Hospital, Inova Health System, Falls Church, VA, USA; e Department of Surgery, Johns Hopkins University School of Medicine, Baltimore, MD, USA

**Keywords:** Epidemiology, *Clostridiodes difficile*, microbiome, infants, children

## Abstract

There has been an increase in the prevalence of Clostridioides difficile (C. diff) causing significant economic impact on the health care system. Although toxigenic C. diff carriage is recognized in infancy, there is limited data regarding its longitudinal trends, associated epidemiolocal risk factors and intestinal microbiome characteristics. The objectives of our longitudinal cohort study were to investigate temporal changes in the prevalence of toxigenic C.diff colonization in children up to 2 years, associated epidemiological and intestinal microbiome characteristics. Pregnant mothers were enrolled prenatally, and serial stool samples were collected from their children for 2 years. 2608 serial stool samples were collected from 817 children. 411/817 (50%) were males, and 738/817 (90%) were born full term. Toxigenic C.diff was detected in 7/569 (1%) of meconium samples, 116/624 (19%) of 2 m (month), 221/606 (37%) of 6 m, 227/574 (40%) of 12 m and 18/235 (8%) of 24 m samples. Infants receiving any breast milk at 6 m were less likely to be carriers at 2 m, 6 m and 12 m than those not receiving it. (*p* = 0.002 at 2 m, *p* < 0.0001 at 6 m, *p* = 0.022 at 12 m). There were no robust differences in the underlying alpha or beta diversity between those with and without toxigenic C. diff carriage at any timepoint, although small differences in the relative abundance of certain taxa were found. In this largest longitudinal cohort study to date, a high prevalence of toxigenic C. diff carrier state was noted. Toxigenic C. diff carrier state in children is most likely a transient component of the dynamic infant microbiome.

## Introduction

C*lostridioides difficile* (*C. diff*) is a Gram-positive, anaerobic spore forming pathogen that causes a spectrum of manifestations from asymptomatic carrier state to diarrhea, fulminant colitis, toxic megacolon, sepsis, and death.^1^ Epidemiological studies have shown an increasing prevalence of toxigenic *C. diff* infection (CDI) in both children and adults causing significant morbidity and economic impact on the health care system.^2–4^ Infants represent a unique cohort as cross-sectional studies have shown a high *C. diff* carrier state in infancy around 25-70% that approaches the adult carrier rate of 3% at around 8 years of age; although many of these studies did not differentiate toxigenic strains.^5–12^ Thus infants can be a potential reservoir for community spread of *C.diff*, specifically if they have disease causing toxigenic strains.^13,14^ Several factors that can detrimentally impact the intestinal microbiome have been associated with *C.diff* carriage in infancy.^14–21^ These include maternal factors (use of prenatal/perinatal antibiotics, mode of delivery) and infant factors (earlier gestational age, mode of feeding, hospitalizations, use of antibiotics and proton pump inhibitors).^14,22,23^ Intestinal dysbiosis has been associated with CDI and asymptomatic carriage in older children and adults.^21,24,25^ However there is paucity of data on the chronological drifts of toxigenic *C.diff* carriage during infancy, associated microbiome characteristics and epidemiological risk factors. Although previous studies have shown conflicting associations of *C.diff* carriage in infancy with atopic sensitization, there is limited understanding of potential long term health consequences.^14–18–26–28^

The aims of this study was to investigate the longitudinal trends in the prevalence of toxigenic *C.diff* carrier state through infancy to 2 years of age and identify associated clinical, demographic and intestinal microbiome characteristics.

## Results

### Subject demographics and clinical characteristics

A total of 2608 serial stool samples were collected from 817 children until around the age of 24 m from April 2018 to August 2021. 411/817 (50%) infants were males, 499/817 (61%) were born vaginally, and 738/817 (90%) were full term (>37 weeks of gestation) (Table 1). The study group had a diverse racial and ethnic background (Table 1). 170/817 (21%) and 456/817 (56%) mothers had received prenatal and perinatal antibiotics respectively.

**Table 1.** Distribution of clinical and demographic variables ‘positive group’ (toxigenic clostridioides difficile positive) versus negative group (non-toxigenic clostridioides difficile positive/clostridioides difficile negative) at 2, 6, 12 and 24 months of age.

	Toxigenic *Clostridioides difficile* at 2 months			Toxigenic *Clostridioides difficile* at 6 months			Toxigenic *Clostridioides difficile* at 12 months			Toxigenic *Clostridioides difficile* at 24 months		
	Positive (*n* = 116)	Negative (*n* = 508)	p- value	Positive (*n* = 221)	Negative (*n* = 385)	p- value	Positive (*n* = 227)	Negative (*n* = 347)	p- value	Positive (*n* = 18)	Negative (*n* = 217)	p- value
**Gender** Male Female	65 (56%) 51 (44%)	259 (51%) 247 (49%)	0.345	114 (52%) 105 (48%)	184 (48%) 197 (51%)	0.375	111 (49%) 116 (51%)	170 (49%) 174 (50%)	0.984	9 (50%) 9 (50%)	110 (51%) 105 (48%)	0.924
**Maternal ethnicity** Hispanic Non-Hispanic	23 (20%) 93 (80%)	99 (20%) 408 (80%)	0.941	44 (20%) 177 (80%)	78 (20%) 305 (79%)	0.742	40 (18%) 187 (82%)	91 (26%) 255 (74%)	**0.015^a^**	3 (17%) 15 (83%)	34 (16%) 183 (84%)	0.911
**Maternal race** Caucasian Asian African American or Black American Indian Native Hawaiian More than one race Other	86 (74%) 9 (8%) 9 (8%) 1 (1%) 0 1 (1%) 10 (9%)	349 (69%) 74 (15%) 20 (4%) 6 (1%) 0 25 (5%) 33 (7%)	**0.049^a^**	161 (73%) 19 (9%) 11 (5%) 2 (1%) 1 (1%) 11 (5%) 16 (7%)	272 (71%) 55 (14%) 11 (3%) 3 (1%) 0 17 (4%) 25 (7%)	0.275	164 (72%) 25 (11%) 10 (4%) 2 (1%) 0 11 (5%) 14 (6%)	252 (73%) 43 (12%) 9 (3%) 3 (1%) 1 (1%) 15 (4%) 23 (7%)	0.883	13 (72%) 2 (11%) 1 (6%) 0 0 2 (11%) 0	160 (74%) 23 (11%) 9 (4%) 4 (2%) 0 10 (5%) 11 (5%)	0.745
**Maternal age in years** (mean, SD)	32.6±4.76	33.2 ± 4.43	0.195	33.09 ± 4.84	33.48 ± 4.42	0.307	33.17 ± 4.45	33.19 ± 4.61	0.950	34.61 ± 3.74	33.53 ± 4.45	0.322
**Maternal weight gain in pregnancy^c^** Less than recommended Recommended More than recommended	15 (13%) 35 (30%) 65 (56%)	87 (17%) 171 (34%) 246 (48%)	0.291	39 (18%) 56 (25%) 124 (56%)	66 (17%) 136 (35%) 177 (46%)	**0.025a**	37 (16%) 59 (26%) 129 (57%)	66 (19%) 125 (36%) 152 (44%)	**0.008a**	4 (22%) 3 (17%) 11 (61%)	40 (18%) 67 (31%) 109 (50%)	0.442
**Full term gestational age** Yes No	109 (94%) 7 (6%)	461 (91%) 45 (9%)	0.315	196 (89%) 24 (11%)	352 (91%) 29 (8%)	0.169	206 (91%) 20 (9%)	310 (89%) 34 (10%)	0.680	15 (83%) 3 (17%)	192 (89%) 24 (11%)	0.478
**Gestational age in weeks** (mean, SD)	38.6±-1.3	38.4 ± 1.7	0.421	38.3 ± 1.7	38.5 ± 1.5	0.130	38.4 ± 1.7	38.4 ± 1.9	0.900	38.2 ± 1.6	38.2 ± 2.1	0.971
**Mode of delivery** Vaginal Caesarean	66 (57%) 50 (43%) ‘	320 (63%) 188 (37%)	0.222	137 (62%) 84 (38%)	229 (60%) 154 (40%)	0.594	141 (62%) 86 (38%)	215 (62%) 13 (38%)	0.995	11 (61%) 7 (39%)	127 (59%) 90 (42%)	0.830
**Prenatal antibiotics** Yes No	21 (18%) 91 (78%)	106 (21%) 394 (78%)	0.563	41 (19%) 175 (79%)	81 (21%) 294 (76%)	0.448	52 (23%) 171 (75%)	66 (19%) 276 (80%)	0.250	2 (11%) 16 (89%)	43 (20%) 174 (80%)	0.367
**Peripartum antibiotics** Yes No	73 (63%) 42 (36%)	274 (54%) 229 (45%)	0.079	119 (54%) 101 (46%)	158 (41%) 219 (57%)	0.341	123 (54%) 101 (45%)	195 (56%) 148 (43%)	0.649	12 (67%) 5 (28%)	128 (59%) 87 (40%)	0.369
**Infant antibiotic use by 2 months** Yes No	3 (3%) 107 (92%)	23 (5%) 466 (92%)	0.358	7 (3%) 158 (72%)	14 (4%) 280 (73%)	0.798	6 (3%) 181 (80%)	10 (3%) 237 (68%)	0.645	2 (11%) 12 (67%)	9 (4%) 161 (74%)	0.172
**Infant antibiotic use by 6 months** Yes No				21 (10%) 192 (87%)	27 (7%) 344 (90%)	0.274	15 (7%) 168 (74%)	22 (6%) 249 (72%)	0.976	2 (11%) 14 (78%)	19 (9%) 163 (75%)	0.797
**Infant antibiotic use by 12 months** Yes No							42 (19%) 179 (79%)	61 (18%) 28 (81%)	0.738	5(28%) 9 (50%)	44 (20%) 148 (68%)	0.277
**Infant antibiotic use by 24 months** Yes No										3 (17%) 15 (83%)	8 (13%) 184 (85%)	0.679
**Number of breast milk feeds/week at 6 months** (mean, SD)	22.3 ± 22.7 (*n* = 82)	27.8 ± 22.8 (*n* = 339)	**0.053a**	19.8 ± 20.7 (*n* = 154)	30.9 ± 22.9 (*n* = 279)	**0.001a**	25.8 ± 23.8 (*n* = 169)	29.6 ± 23.08 (*n* = 229)	0.113	30.5 ± 25.01 (*n* = 16)	28.4 ± 22.8 (*n* = 184)	0.735
**Number of breast milk feeds/week at 12 months** (mean, SD)	11.9 ± 16.6 (*n* = 72)	12.7 ± 17.2 (*n* = 325)	0.725	8.77 ± 14.52 (*n* = 142)	14.94 ± 17.87 (*n* = 260)	**<0.001a**	11.2 ± 16.1 (*n* = 169)	13.3 ± 17.7 (*n* = 247)	0.219	11.9 ± 21.1 (*n* = 17)	11.8 ± 16.8 (*n* = 167)	0.983
**Any breast milk at 6 months** Yes No	48 (41%) 34 (29%)	256 (50%) 84 (17%)	**0.002a**	94 (43%) 60 (27%)	219 (57%) 61 (16%)	**<0.001a**	114 (50%) 56 (25%)	177 (51%) 52 (15%)	**0.022a**	12 (67%) 4 (22%)	140 (65%) 45 (21%)	0.951
**Any breast milk at 12 months** Yes No	30 (26%) 42 (36%)	144 (28%) 184 (36%)	0.728	44 (20%) 98 (44%)	135 (35%) 128 (33%)	**<0.001a**	67 (30%) 102 (45%)	113 (33%) 137 (40%)	0.259	5 (28%) 12 (67%)	69 (32%) 98 (45%)	0.340
**Daycare at 6 months** Yes No	35 (30%) 43 (37%)	137 (27%) 187 (37%)	0.813	74 (34%) 77 (35%)	98 (26%) 174 (45%)	**0.025a**	77 (34%) 81 (36%)	89 (26%) 139 (40%)	0.117	8 (44%) 10 (56%)	89 (41%) 90 (42%)	0.867
**Daycare at 12 months** Yes No	31 (27%) 34 (29%)	121 (24%) 178 (35%)	0.284	63 (29%) 65 (29%)	89 (23%) 154 (40%)	**0.008a**	68 (30%) 89 (39%)	95 (27%) 134 (39%)	0.721	7 (39%) 10 (56%)	87 (40%) 67 (31%)	0.228
**Toxigenic carriage at 2 months** Yes No				60 (27%) 109 (49%)	27 (7%) 275 (71%)	**<0.001a**	45 (20%) 144 (63%)	29 (8%) 227 (65%)	**<0.001a**	2 (11%) 13 (72%)	32 (15%) 141 (65%)	0.618
**Toxigenic carriage at 6 months** Yes No							90 (40%) 98 (43%)	79 (23%) 197 (57%)	**<0.001a**	4 (22%) 13 (72%)	68 (31%) 121 (56%)	0.302
**Toxigenic carriage at 12 months** Yes No										6 (33%) 9 (50%)	77 (36%) 114 (53%)	0.980

a American College of Obstetricians and Gynecologists.

b Missing/Unknown values are not reported in the table.

c Significant p values.

### Epidemiology of toxigenic C. diff and clinical associations

**Figure 1.**
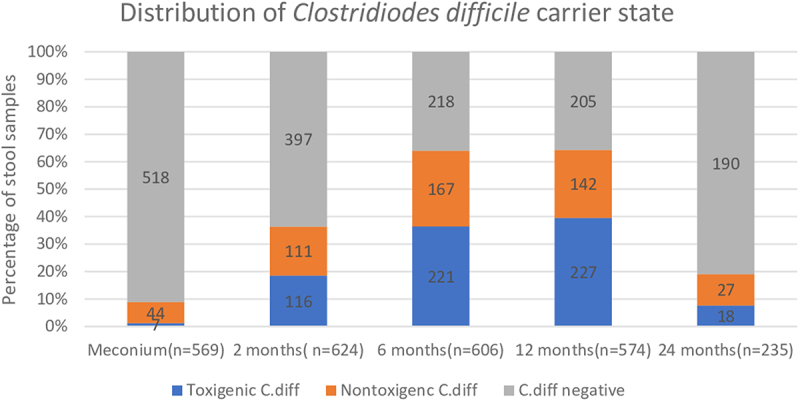
Longitudinal distribution of toxigenic clostridioides difficile carrier state from birth to 2 years of age.

The prevalence of toxigenic *C.diff* at each timepoint was evaluated after longitudinal stool samples were collected. 7/569 (1%), 116/624 (19%), 221/606 (37%), 227/574 (40%) and 18/235 (8%) of meconium, 2 m, 6 m,12 m, 24 m were positive respectively (Figure 1). Longitudinal stool samples at 2 m, 6 m and 12 m were available in 181/817 (22%) infants and 27/181 (15%) were ‘always positive’. The inter-quartile ranges for CD 16s rRNA, tcdA, tcdB primer/probe sets were 20.32-31.2 (75% of reported detections with Ct/cycle threshold values under 31.20), 23.98-32.36 (75% of reported detections with Ct values under 32.36) and 24.24-32.76 (75% of reported detections with Ct values under 32.76) respectively. No infant was clinically diagnosed with CDI.

Clinical and demographic variables (as described in methods) were then studied for any significant association. Infants with non-Hispanic mothers were more likely to be positive when compared to those with Hispanic mothers at 12 m (42% in non-Hispanic group versus 31% in Hispanic group; *p* = 0.015, chi-square). Other demographic characteristics were similar between the two groups. Infants born to mothers with more than recommended weight gain during pregnancy (per American College of Obstetricians and Gynecologists guidelines) were more likely to be positive at 6 m and 12 m (*p* = 0.025 and 0.008 at 6 m and 12 m respectively).

Infants receiving any breast milk at 6 m were less likely to be positive at 2 m, 6 m and 12 m than those not receiving it (16% in breast milk group versus 29% in no breast milk group at 2 m, *p* = 0.002; 30% in breast milk group versus 50% in no breast milk group at 6 m, *p* < 0.001; 39% in breast milk group versus 52% in no breast milk group at 12 m, *p* = 0.022, chi-square). This was directly proportional to the number of times infants received breast milk per week (Table 1).

Infants attending daycare at 6 m were more likely to be positive (43% in daycare group versus 31% in no daycare group, *p* = 0.025, chi-square). There was no significant difference in carrier state with mode of delivery, prenatal or peripartum antibiotic use, child gender, gestational age, and infant antibiotic use.

Infants positive at 2 m were more likely to test positive at 6 m (69% in positive versus 28% in negative; *p* < 0.001) and 12 m (61% in positive versus 39% in negative, *p* < 0.001, chi-square). Infants positive at 6 m were more likely to be positive at 12 m (53% in positive versus 33% in negative, *p* < 0.001, chi-square).

Infants who were ‘always positive’ received more formula feeds per week when compared to ‘always negative’ at 12 m (mean = 13.0 and 7.02 in always positive and always negative respectively, *p* = 0.004, two sample t-test) (Table 2).

**Table 2.** Distribution of clinical and demographic variables between ‘always positive’ toxigenic Clostridioides difficile (positive at 2 months, 6 months, 12 months) versus ‘always negative’ groups (negative at 2 months, 6 months and 12 months).

	“Always positive” for toxigenic *Clostridioides difficile at 2,6 and 12 months* (*n* = 27)	“Always negative” for toxigenic *Clostridioides difficile at 2,6 and 12 months* (*n* = 154)	p-value
**Gender** Male Female	16 (59%) 11 (41%)	83 (54%) 71 (46%)	0.605
**Maternal ethnicity** Hispanic Non-Hispanic	2 (7%) 25 (93%)	25 (16%) 129 (84%)	0.235
**Maternal race** Caucasian Asian African American American Indian Native Hawaiian More than one race Other	21 (78%) 3 (11%) 1 (4%) 0 0 1 (4%) 1 (4%)	116 (75%) 23 (15%) 2 (1%) 2 (1%) 0 8 (5%) 3 (2%)	0.872
**Maternal age in years** (mean, SD)	32.8 ± 5.2	33.7 ± 4.5	0.3197
**Maternal weight gain in pregnancy (ACOG)b** Less than recommended Recommended More than recommended	2 (7%) 6 (22%) 18 (67%)	25 (16%) 56 (36%) 71 (46%)	0.101
**Term gestational age** Yes No	25 (93%) 2 (7%)	130 (84%) 16 (10%)	0.632
**Mode of delivery** Vaginal Caesarean	16 (59%) 11 (41%)	86 (56%) 68 (44%)	0.741
**Prenatal antibiotics** Yes No	3 (11%) 23 (85%)	25 (16%) 128 (83%)	0.545
**Peripartum antibiotics** Yes No	17 (63%) 10 (37%)	97 (63%) 57 (37%)	0.06
**Infant antibiotic course by 2 months** Yes No	1 (4%) 26 (96%)	9 (6%) 140 (91%)	0.629
**Infant antibiotic use by 6 months** Yes No	0 27 (100%)	10 (7%) 141 (92%)	0.168
**Infant antibiotic use by 12 months** Yes No	6 (22%) 20 (74%)	27 (18%) 125 (81%)	0.519
Infant antibiotic use at 24 months Yes No	1 (4%) 21 (78%)	12 (8%) 96 (62%)	0.349
**Number of breast milk feeds/week at 6 months** (mean, SD)	25.8 ± 22 (*n* = 25)	32.6 ± 23.8 (*n* = 123)	0.188
**Number of breast milk feeds/week at 12 months** (mean, SD)	13.6 ± 15.5 (*n* = 23)	19.38 ± 28.4 (*n* = 122)	0.353
**Any Breast milk at 6 months** Yes No	17 (63%) 8 (30%)	100 (65%) 23 (15%)	0.136
**Any Breast milk at 12 months** Yes No	12 (45%) 11 (41%)	66 (43%) 56 (36%)	0.865
**Number of formula feeds/week at 6 months** (mean, SD)	8.9 ± 13.4 (*n* = 24)	9.8 ± 14.6 (*n* = 123)	0.789
**Number of formula feeds/week at 12 months** (mean, SD)	13.0 ± 15.5 (*n* = 23)	7.02 ± 12.5 (*n* = 122)	**0.042a**
**Daycare at 6 months** Yes No	11 (41%) 11 (41%)	43 (28%) 75 (49%)	0.441
**Daycare at 12 months** Yes No	10 (37%) 10 (37%)	40 (26%) 71 (46%)	0.236

^a^Significant *p* values.

^b^American College of Obstetricians and Gynecologists.

^c^Missing/Unknown values are not reported in the table.

### Microbiome associations with toxigenic C. diff carrier state

**Figure 2.**
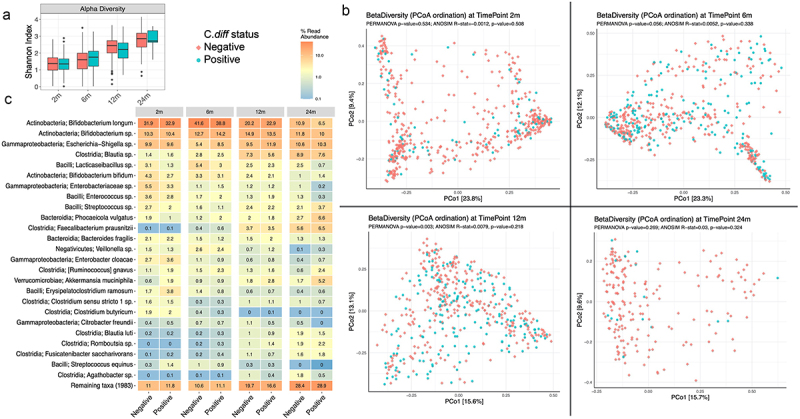
Microbiome characteristics in ‘positive group’ (toxigenic clostridioides difficile positive) versus negative group (non-toxigenic clostridioides difficile positive/clostridioides difficile negative) at 2, 6, 12 and 24 months of age. A- distribution of alpha diversity (shannon index); B- Principal coordinate analysis plot for beta diversity (bray-curtis index), C-Heat map diagram of the relative abundance of taxa by carrier state.

Microbiome data was available in 514/624 (82%), 509/606 (84%), 466/574 (81%) and 207/235 (88%) of 2 m, 6 m,12 m and 24 m samples respectively. The visual community diversity explorations and statistical diversity analyses showed no significant differences in the alpha diversity or beta diversity at 2 m, 6 m, and 24 m between positive and negative groups (Figure 2). Although statistically significant differences were detected in community diversities between groups at 12 m (ANOVA on Alpha diversity p = 0.001 and PERMANOVA on Beta diversity p = 0.003), further community explorations showed minimal factor contribution (ANOSIM effect size~0.8%, *p* >>0.5), location-based separation (PERMDISP F-value = 0.5, *p* >> 0.5) and no clear visual separation of the communities (Figure 2). Significant abundance changes were detected in a few taxa between the 2 groups (supplementary Figure S1). There was an overall decrease in *Lacticaseibacillus species and an* increase in *Proteus mirabilis* in the positive group at 12 m.

**Figure 3.**
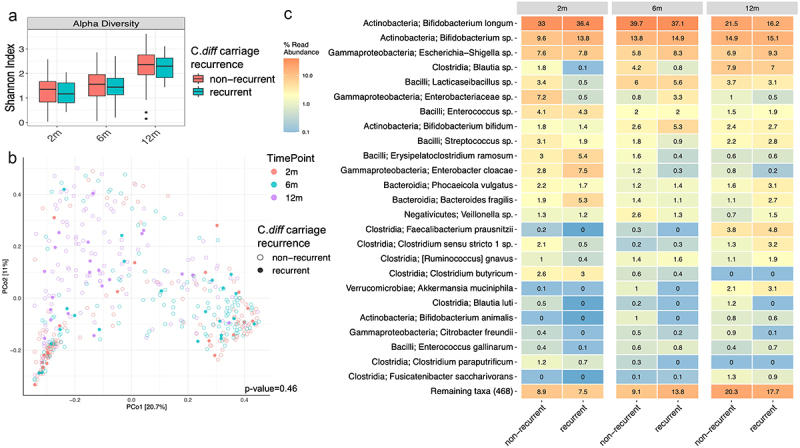
Microbiome characteristics in ‘recurrent carriage group’ (defined as positive for toxigenic clostridioides difficile at 2 or more timepoints) versus ‘non-recurrent carriage group’ (rest of the subjects) at 2, 6, 12, 24 months. A- distribution of alpha diversity (shannon index); B- Principal coordinate analysis plot for beta diversity (bray-curtis index); C- heat map diagram of the relative abundance of taxa by carrier state.

In the longitudinal cohort with samples at all time points (2 m, 6 m and 12 m) subjects were divided into two cohorts for microbiome analysis: ‘recurrent carriage group’ defined as positive at 2 or more timepoints and the rest were included in ‘non-recurrent carriage group’. There was no significant difference in the alpha diversity, beta diversity and relative abundance of most taxa between the two groups at all timepoints (Figure 3). There was a significant decrease in the relative abundance of some taxa (*Bifidobacterium, Lacticaseibacillus, Lactobacillus*, *Veilonella*, *Streptococcus*, certain *Bacteriodes* and *Enterobacteriaceae species)* and increase in some Firmicutes (*Eisenbergiella* species) in the recurrent carriage group (Supplementary Figure S2).

There were no significant differences in the alpha diversity, beta diversity or relative abundance of most taxa between infants exclusively on breast milk versus formula at 6 m when adjusted for colonization status (Supplementary Figure S3). *Bifidobacterium* and *Lacticaseibacillus species* were most differentially abundant in negative infants on breast milk and least abundant in positive infants on formula. Firmicutes (*Fusicatenibacter saccharivorans*) and Gammaproteobacteria (*Escherichia-Shigella species*) were most differentially abundant in positive formula fed babies (Supplementary Figure S4)

## Discussion

In the largest longitudinal study to date on the epidemiology of toxigenic *C.diff* carriage in children up to 2 years of age and its intestinal microbiome associations, a high prevalence rate was noted with a peak of 40% at 12 months. Infants receiving breast milk were less likely to be carriers and there were no major associated changes in microbiome diversity.

A recent metanalyses on the epidemiology of *C.diff* showed a pooled prevalence rate of toxigenic *C.diff* at 14% around 6-12 m.^5^ Even though the longitudinal trends for toxigenic carrier state in our study were consistent with the findings from previous studies, we had a much higher prevalence rate at 36% −40% at 6-12 m.^5–7-9–19-29^ These differences could be due to the increase in its prevalence with time, geographic variations in distribution and differences in study design (most of these studies were cross sectional with smaller sample size) and *C.diff* detection techniques used (validated detection of toxin gene by PCR utilized in our study is the most sensitive).^30–32^ The very low prevalence of *C.diff* in meconium samples and the steady increase during rest of infancy suggests that it is most likely environmentally acquired rather than maternally transmitted at birth; however maternal *C.diff* status at delivery was not obtained to confirm this hypothesis.^14^ Infants positive in earlier samples were more likely to again test positive. This implies that toxigenicity has a propensity for persistence through infancy once acquired. Thus infants may act as a significant reservoir for community spread of toxigenic *C.diff* given its high prevalence rate and tendency to persist after initial colonization.

Intestinal dysbiosis with an overall decrease in microbial diversity and richness has been associated with asymptomatic colonization and CDI in older children and adults.^21,24,25^ These changes include an increase in Firmicutes/Bacteriodes ratio, Proteobacteria (Enterobacteriaceae/*Enterococcus*), *Lactobacillus* and *Veilonella* species and a decrease in *Lachnospiraceae*, Ruminococcaceae, Clostridium IV and XIV clusters, *Faecalibacterium prausnitzii* and *Bifidobacterium* species.^20,21,33^ A limited number of cross-sectional studies investigating microbiome changes in colonization during infancy have described an increase in Firmicutes, *Klebsiella* species and decrease in *Bifidobacterium* species.^19,23,29^ It is not clearly understood if *C. diff* is a mediator of these changes or just a part of the dysbiotic state. To our knowledge no longitudinal studies to date have examined intestinal microbiome associations with toxigenic *C.diff* in infancy. We noted significant differences in the relative abundance of certain taxa similar to previous studies, however, without any robust changes in microbial diversity. A novel finding was a consistent decrease in *Lacticaseibacillus* species in carrier state. Contrary to previous adult studies, an increase in Enterobacteriaceae or *Veilonella* species was not noted with toxigenicity.^24,34,35^ Since there were no distinct microbial signatures causing robust changes in diversity with carriage, it is most likely a transient component in the dynamic infant microbiome as it transitions toward the relatively stable adult microbiome.

Our study noted that infants receiving breast milk during infancy were less likely to be positive. Breast milk has several antimicrobial factors including human milk oligosaccharides, lysozymes, secretory IgA and a unique microbiome (mostly commensal bacteria; *Bifidibacterium* and *Lactobacillus)* that possibly offer protection against colonization.^36–38^ Drall et al noted that exclusively breast-fed babies colonized with *C.diff* at 3 m had a different gut microbial profile resembling that of formula-fed babies and greater alpha-diversity than those not colonized.^23^ However in our study there was no significant difference in microbial diversity between infants exclusively on breast milk versus formula at 6 m when adjusted for carrier state. These contradicting findings could be due to differences in study design (subject age, diet and study methods as colonization status without toxigenicity was analyzed in Drall et al study). Our findings suggest that even though breast milk modulates the intestinal microbiome, the differences in carrier state with mode of feeding is less likely mediated through these changes.

A higher incidence of toxigenicity was noted in subjects with non-Hispanic mothers at 12 months. This aligns with findings from a recent case-control study that demonstrated higher risk of CDI in non-Hispanic children.^6^ The higher positive rate in infants of mothers with more than recommended weight gain during pregnancy could possibly be from maternal transmission postnatally as adult studies have shown association between obesity and CDI.^39^ The higher positive rate in infants attending day care again suggests that it is most likely environmentally acquired.^40,41^ Infant use of antibiotics and prolonged hospitalizations have been associated with carrier state and infection.^14,16^ Our study group consisted mostly of term infants with limited use of antibiotics and hence might not have captured a potential association.

Despite the high carrier state, there was no documented clinical CDI in our study group irrespective of feeding method and other clinical factors. The absence of toxigenic *C.diff* receptors is one of the postulated reasons; however this hypothesis is based on animal studies with small sample size.^42,43^ The overall features of the developing infant microbiome which differs from adult microbiome in structure and composition are another possible protective factor against CDI.^44–46^ Several studies have demonstrated differences in gut metabolomic profiles with CDI in adults characterized by a depletion of short chain fatty acid (SCFA) and secondary bile acids.^47,48^ The infant gut has a different SCFA metabolomic profile based on feeding (higher levels of acetate in infants receiving breast milk and prebiotic containing formula) and has a bile acid profile that is initially predominated by primary bile acids which may also be permissive to carriage.^49–51^ However, further studies are needed to elucidate comprehensive characteristics of infant gut microbiome and metabolome in carrier state and its role in protection against CDI. This may offer strategies against CDI in older children and adults. The high prevalence of asymptomatic carriage in infancy reiterates that testing for CDI in this population should be conducted with great caution, as per the IDSA guidelines, due to the possibility of detecting carriage rather than true infection.^52^ Children can develop humoral immune responses against *C.diff* toxin even in the absence of CDI; however the duration of protection from anti-toxin antibodies and its long term health implications need further investigation.^53^

The major strengths of our study included its large sample size, longitudinal study design and attention to toxigenicity that provided a better understanding of the dynamics of *C.diff* carrier state in infancy right from the neonatal period. The major limitation of our study was that it was a single center study and even though we had racially and ethnically diverse group the results of our study may be less generalizable to other regions of the world. Moreover, although this is the first longitudinal study to assess intestinal microbiome associations of toxigenic *C. diff* carriage in infancy, 16S rRNA gene sequencing was used. Utilization of shotgun metagenomic sequencing and metabolomic analysis may have provided further insight into characteristics of the microbiome at species level, in addition to non-bacterial microorganisms, as well as its functional properties.^54^ In addition our study consisted mostly of term infants and hence the prevalence rate may not be reflective of distributions in preterm infants with prolonged hospitalization and more frequent antibiotic exposure.^55,56^

## Conclusions

This study gives novel insight into the longitudinal dynamics of toxigenic *C. diff* carriage in infancy and associated epidemiological, clinical and intestinal microbiome characteristics. Given its high prevalence rates, infants may act as potential community reservoir. Infants receiving breast milk were less likely to be carriers, most likely due to its anti-microbial properties rather than direct effects on the microbiome composition. Colonization in children is not associated with significant changes in microbial diversity and hence it is most likely a transient component of the dynamic infant microbiome.

## Methods

### Study design and sample collection

Mothers were enrolled prenatally with informed consent in a longitudinal, prospective cohort study “The First 1000 Days of Life and Beyond” within the Inova Health System. All experimental protocols were approved by the Inova Health System and WCG Institutional Review Board (Inova protocol #15-1804, WCG protocol #20120204). Serial stool samples were collected from infants starting in the first 2 days after birth (meconium) and at around 2 months(m), 6 m, 12 m, and 24 m of age. All samples except meconium were collected by caregivers at home and mailed back to the lab, using previously validated methods and stored at −80°C until analysis.^57,58^

Demographic information including infant gender, maternal race/ethnicity, pregnancy details including mode of delivery (vaginal delivery versus cesarean section), use of prenatal antibiotics (antibiotics given to mother from conception to 2 days before delivery) and peripartum antibiotics (antibiotics given to mother 2 days before and during delivery), gestational age at delivery, and maternal weight gain during pregnancy were collected through a questionnaire and review of electronic medical records. Details including infant feeding, daycare attendance, use of antibiotics were also collected from parents through a questionnaire.

### DNA extraction and microbiome sequencing

DNA was extracted from stool aliquots using the DNeasy PowerSoil Pro kit (Qiagen, Valencia, CA) following manufacturer’s instructions. Barcoded PCR primers annealing to the V4 region of 16S ribosomal RNA gene were used for library generation (forward primer 5’-GTGYCAGCMGCC-GCGGTAA-3’, reverse primer: 5’-GGACTACN-VGGGTWTCTAAT-3’). Sequencing was performed on the Miseq platform (Illumina, CA, USA) with 2 × 250 base pair paired end reads. Positive controls (DNA sequences) and negative controls (DNA free water) were used.

### qPCR for C. difficile detection

Primers and Taqman-based probes targeting *C. diff* specific 16S rRNA (CD16S rRNA), tcdA (Toxin A), and tcdB (Toxin B) genes were used (Invitrogen, MA, USA) on extracted DNA.^31^ Samples were run in duplicate for each assay and qPCR was performed in 384-well optical plates on ABI QuantStudio 6 Flex Real-Time PCR System (ThermoFisher, CA, USA). Target detection was considered positive with conclusive amplification curves in both replicates of a given primer/probe set; mean Ct values over 46 were considered negative (Ct > 50 was considered negative by the reference study and we lowered this cutoff to>46). Samples positive for CD16S rRNA but negative for both tcdA and tcdB were designated as non-toxigenic *C. diff*, samples positive for CD16S rRNA and either tcdA, tcdB, or both, were designated as positive for toxigenic *C. diff* and samples with no target detection for all three primer probe sets were designated as negative for *C. diff.*

### Epidemiological and statistical analysis

The subjects were divided into following analytical categories: 1) toxigenic C.*diff* positive group hereafter referred to as ‘positive’ versus non-toxigenic *C. diff* positive/*C. diff* negative group hereafter referred to as ’negative’ and 2) ‘always positive’ for toxigenic *C.diff* (positive at time points 2 m, 6 m, 12 m) versus ‘always negative’ for toxigenic *C.diff.*

Correlation between clinical and demographic factors with the prevalence of toxigenic *C.diff* at each timepoint was evaluated using the chi-square test and two-sample t-test for categorical and continues variables respectively.

### Microbiome analysis

Meconium samples were not included due to low *C.diff* and bacterial DNA contents. Amplicon sequence variants (ASV) were generated by denoising and merging reads with DADA2 v1.20.0 and subsequent taxonomic assignment with rdp and the SILVA v138.1 database.^59,60^ ASVs unable to be identified by SILVA were further queried against NCBI’s 16S ribosomal RNA database using BLAST+ v2.12.0.^61^ Sample read depth analysis and filtering were performed with R statistical language in R Studio (tidyverse, ampvis2, vegan, ggplot2).^62–65^ Samples with read abundance<15000 reads and ASVs with abundance<15 reads were excluded from downstream analysis. The 16S rRNA sequence data of qualified samples are available in NCBI Sequence Read Archive (SRA), PRJNA851805.

Community differences were explored visually and statistically between positive and negative groups with the help of R packages.^66,67^ Alpha diversity was explored using Shannon index and groups were compared statistically with ANOVA and Wilcoxon tests. Beta diversity was explored using Bray-Curtis dissimilarity matrix, distances visualized with PCoA and community contrasts explored statistically with PERMANOVA, ANOSIM and PERMDISP (where applicable). Differential abundance testing on species level taxonomy was performed using DESeq2 contrasting the positive with negative samples and p-values adjusted by the Benjamini-Hochberg method for multiple comparisons.^68^

## Supplementary Material

Supplemental Material

## Data Availability

The data that support the findings of this study are openly available in https://www.ncbi.nlm.nih.gov/bioproject/PRJNA851805 [ncbi.nlm.nih.gov]
