# Supplementary figures and images for: Epidemiological and microbiome associations of Clostridioides difficile carriage in infancy and early childhood
Source: Gut Microbes. 2023 Apr 25;15(1):2203969. doi: 10.1080/19490976.2023.2203969 (PMC10132246; doi:10.1080/19490976.2023.2203969)

Cdf\_status ~adj4~ TimePoint

Cdf\_status Healthy ToxCDF

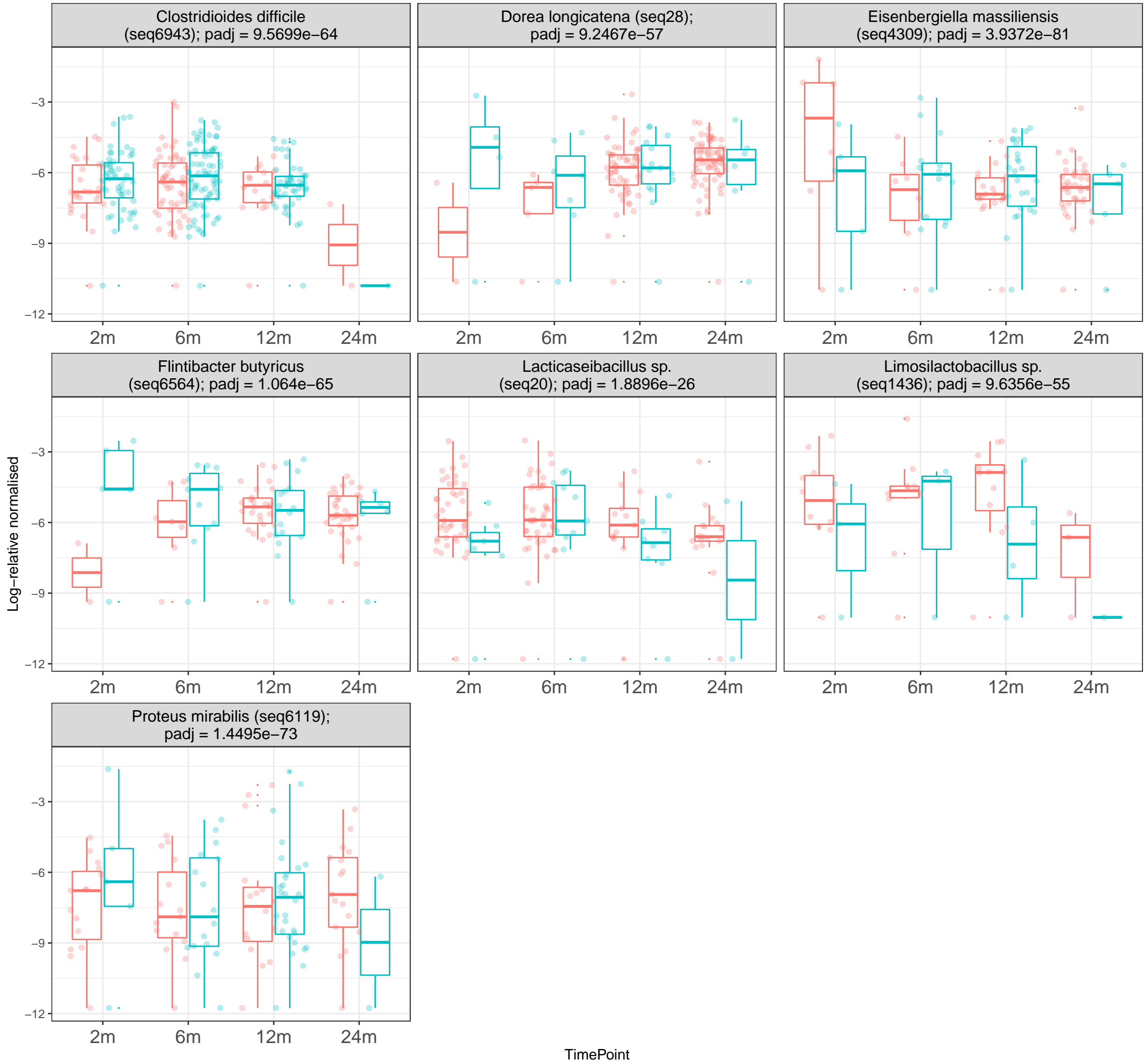

Supplement: Supplemental Material [file KGMI_A_2203969_SM8594.zip › Supplemental Material/Supplementary Figure 1.pdf]

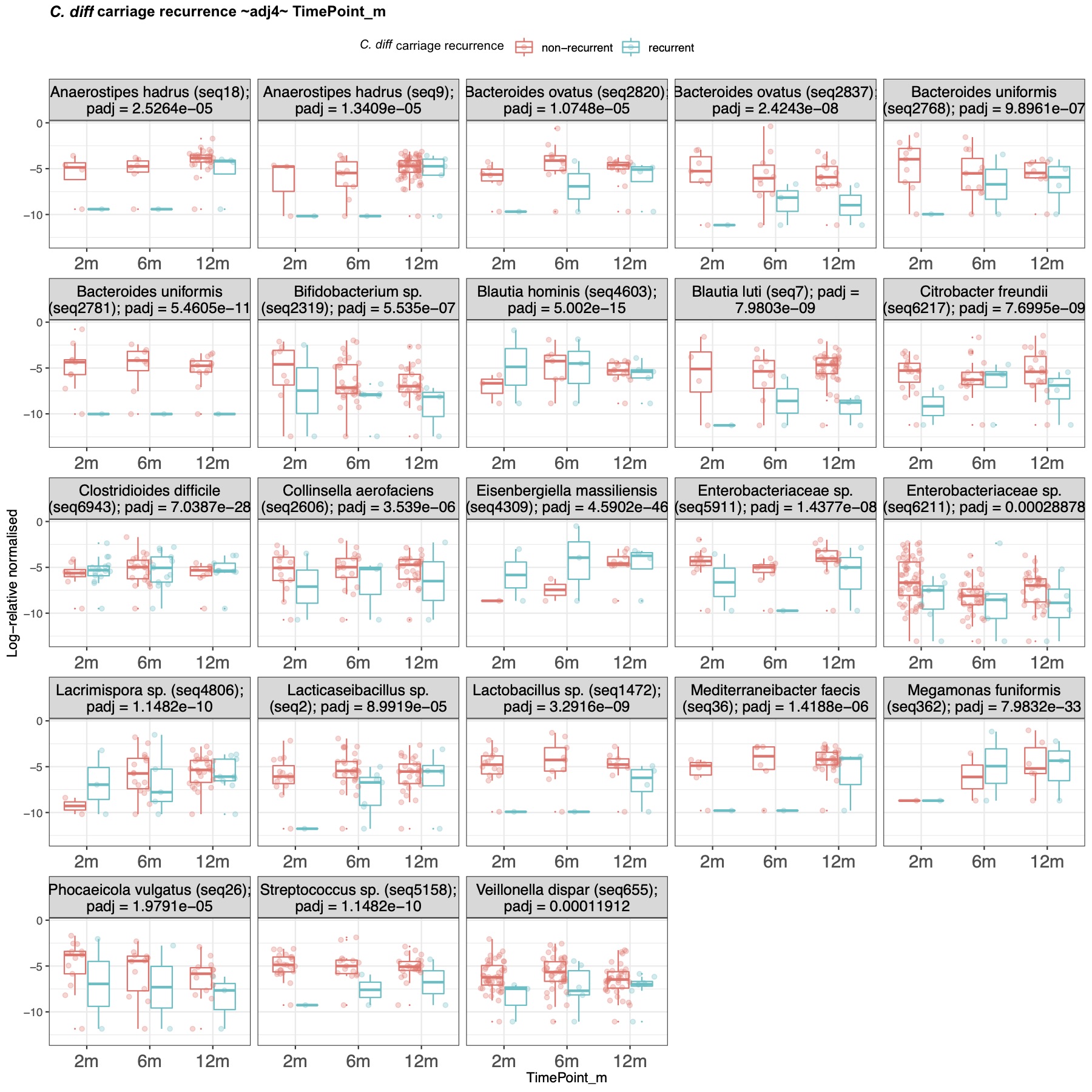

Supplement: Supplemental Material [file KGMI_A_2203969_SM8594.zip › Supplemental Material/Supplementary Figure 2 2123.jpg]

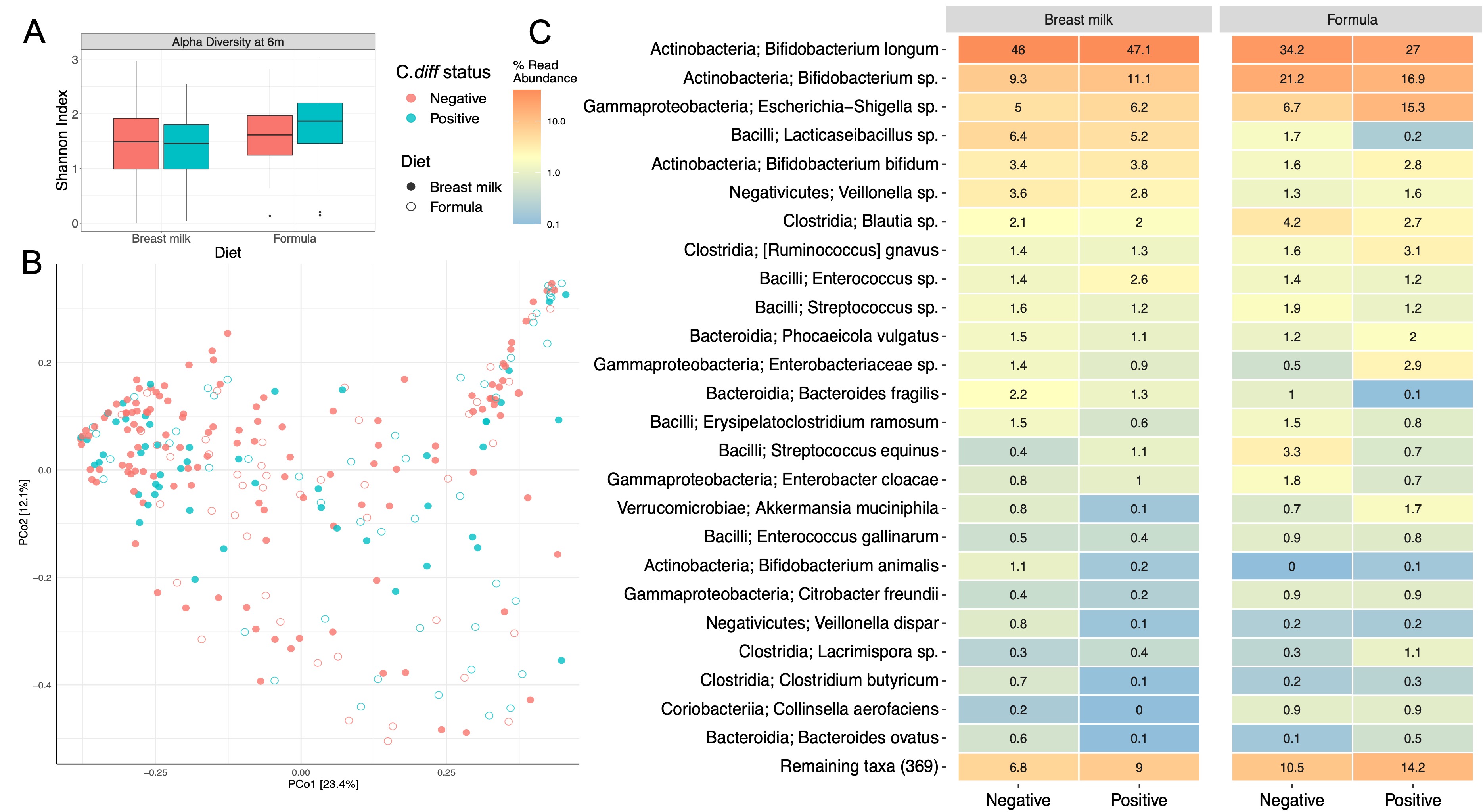

Supplement: Supplemental Material [file KGMI_A_2203969_SM8594.zip › Supplemental Material/Supplementary Figure 3.jpg]

Significance of Cdf\_status within each Diet

Cdf\_status Negative Positive

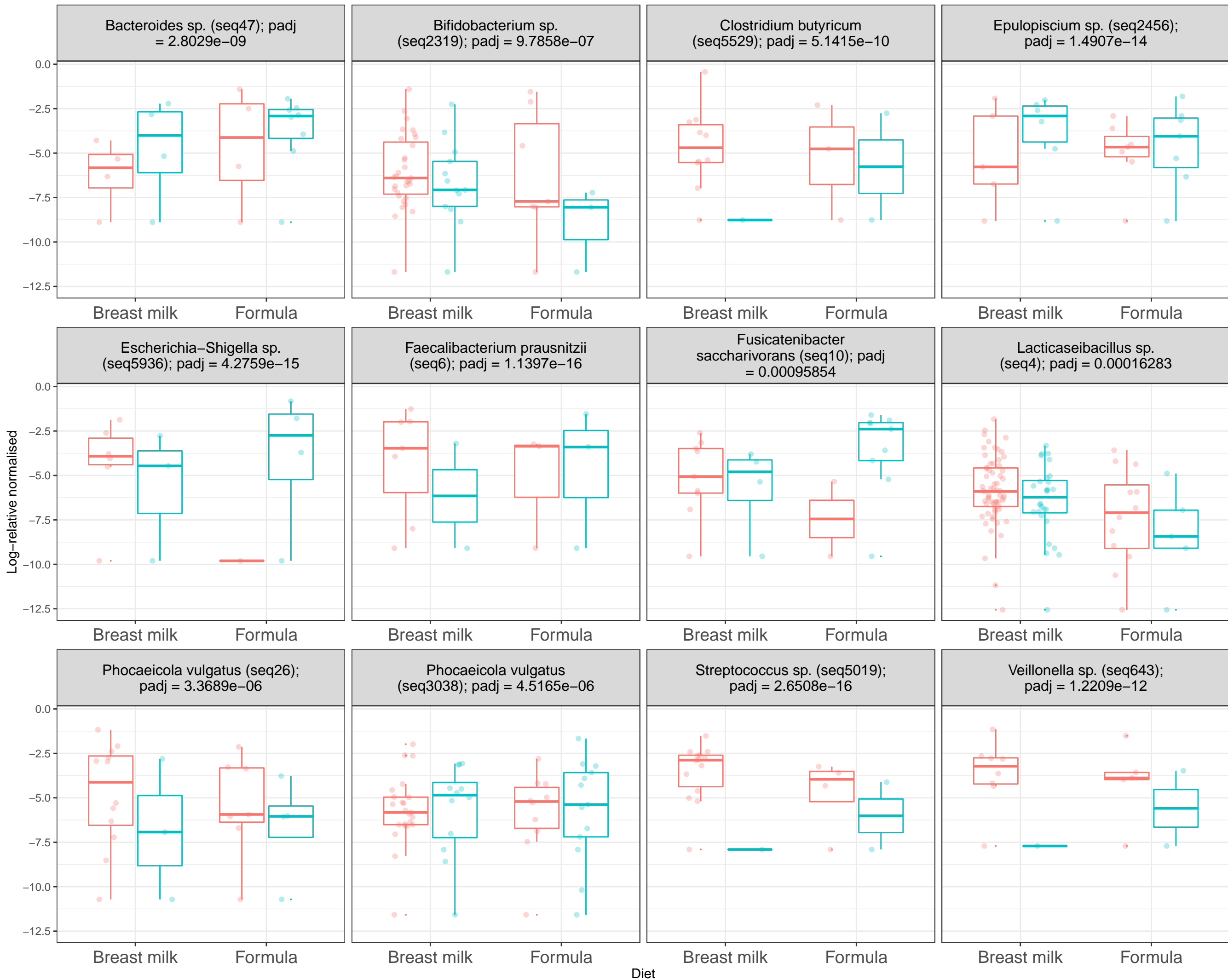

Supplement: Supplemental Material [file KGMI_A_2203969_SM8594.zip › Supplemental Material/Supplementary Figure 4.pdf]
